# Highly-efficient laser ablation of copper by bursts of ultrashort tuneable (fs-ps) pulses

**DOI:** 10.1038/s41598-019-48779-w

**Published:** 2019-08-22

**Authors:** Andrius Žemaitis, Paulius Gečys, Martynas Barkauskas, Gediminas Račiukaitis, Mindaugas Gedvilas

**Affiliations:** 1 Center for Physical Sciences and Technology, Savanoriu Ave. 231, LT-02300 Vilnius, Lithuania; 2 Light Conversion Ltd., Keramiku st. 2B, LT-10233 Vilnius, Lithuania

**Keywords:** Surface patterning, Laser material processing

## Abstract

Ultrashort pulse laser, capable of varying pulse duration between 210 fs and 10 ps and producing a burst of pulses with an intra-burst pulse repetition rate of 64.5 MHz (time distance between pulses 15.5 ns), was used to investigate the ablation efficiency of the copper. The study on ablation efficiency was done for various numbers of pulses per burst between 1 and 40. The increase in the ablation efficiency by 20% for 3 pulses per burst compared to a non-burst regime was observed. The comparison was made between the beam-size optimised regimes. Therefore, the real advantage of the burst regime was demonstrated. To the best of our knowledge, we report the highest laser milling ablation efficiency of copper of 4.84 µm^3^/µJ by ultrashort pulses at ~1 µm optical wavelength.

## Introduction

Ultrafast laser pulses are already widely used in material processing due to high flexibility and precision, but for competitive industry the processing throughput must always grow^1^. Recently, a lot of interest was shown to the studies of laser burst mode processing. One of the most successful studies demonstrated that bursts of high repetition rate pulses could increase ablation efficiency by introducing ablation-cooled material removal^2^. The study reveals that the ablation-cooled regime for copper starts somewhere between the intra-burst pulse repetition rate of 27 MHz and 108 MHz. The burst regime was also employed to enhance the laser modification efficiency of the transparent materials via accumulation effects^3^. The purpose of our work was to investigate the real advantage of the burst regime by comparing the beam-size optimised single-pulse regime with the beam-size optimised multiple-pulse burst regime, which was never previously demonstrated. The optimisation was done by increasing the spot size to find the maximum ablation efficiency for various pulse numbers per burst and different pulse durations as described in^4^. The idea of the optimal fluence for the highest ablation efficiency was proposed in^5^ and experimentally proven in^6^, where groove ablation was investigated. Studies of the ablation efficiency by changing the pulse energy was examined for various materials like metals^7–9^, semiconductors^10,11^, dielectrics^12^ and biological tissues^13^. When ablation optimisation is done by changing the size of the beam, the ablation rate (volume of the material removed per unit of time) and ablation efficiency (volume of the material removed with a unit of energy) are optimised together. The ultrafast Tunable Acoustic Gradient-Index lens was used to increase the micro-machining efficiency by changing the laser spot size^14^.

In this study, we have used a solid-state laser radiating at the 1030 nm wavelength and producing bursts of pulses with the intra-burst pulse repetition rate of 64.5 MHz. The increase up to 20% in the ablation efficiency for 3 pulses per burst compared to a non-burst regime was observed for all 12 tested pulse durations. The similar increase of copper ablation efficiency for the 3 pulses per burst regime compared to a single pulse regime was demonstrated by the pulse energy optimisation at a fixed beam size and reached improvement of 13%^8^ and 15%^15^. We demonstrate that further increase, leading to the highest ever published laser milling ablation efficiency, can be done by the beam-size-optimisation. The maximum ablation efficiency of 4.8 µm^3^/µJ and ablation rate of 0.18 mm^3^/s (10.5 mm^3^/min) was obtained, with the average optical power of 36 W, 3 pulses per burst and 10 ps pulse duration. In our best knowledge, the highest reported value of laser milling ablation efficiency for copper by ultrashort pulse laser emitting at ~1 µm optical wavelength, did not exceed <3 µm^3^/µJ^8,10,15,16^. The quality of processed surface at the optimal processing parameters for the highest ablation efficiency was evaluated by measuring the surface roughness *R*_a_, and it was 0.4 µm. The optimal processing parameters were used for laser milling of complex 3D surfaces.

## Materials and Methods

### Experimental setup

**Figure 1:**
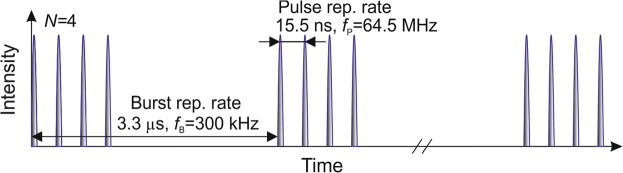
Schematic example of laser burst regime with 4 pulses per burst, the burst repetition rate of 300 kHz and intra-burst (pulse) repetition rate of 64.5 MHz. The distance between bursts is out of the scale compared to pulses distance.

A solid-state laser (Carbide, Light Conversion) with a variable pulse duration in the range of 210 fs–10 ps and radiating at the light wavelength of *λ* = 1030 nm was used in the experiments. The laser had a possibility to emit a burst of a certain number of pulses from 1 (single pulse regime) to 40. The intra-burst pulse repetition rate was 64.5 MHz, corresponding to the time distance between sequential pulses of 15.5 ns (Fig. 1) The burst repetition rate was always fixed at 300 kHz. An average optical power on the sample surface was 36 W. A galvanometer scanner (Intelliscan 14, Scanlab) and F-theta lens with a focal distance of 100 mm were used to scan and focus the laser beam.

### Ablation efficiency characterisation

Rectangular cavities with dimensions of 2 × 1 mm^2^ were engraved into a copper target. Multiple layer scanning was applied to increase the depth and measurement accuracy of cavities profiles. The largest cavity depth reached <80 µm. Cavity depth never exceeded the Rayleigh length of the laser beam. A linear dependence of the cavity depth versus the number of pulses applied was maintained during the experiment. Therefore, the ablation saturation effects were completely avoided, and reliable data were collected for all investigated regimes^17^. The beam scanning speed of 1 m/s and the distance between scanned lines (hatch) of 10 µm were always constant. The maximum laser power at 300 kHz was exploited in the ablation efficiency measurements. However, the laser fluence was varied by changing the spot size of the focused beam. By defocusing the laser beam from 0 mm (focus position) to 5.5 mm, the beam spot radius *w* on the sample surface was varied from 25.9 µm to 82.3 µm, respectively. The ablation efficiencies dependence on laser fluence was investigated for 10 different numbers of pulses in the burst: 1 (single-pulse regime), 2, 3, 4, 5, 6, 10, 20, 30, 40. These experiments were repeated for 12 different pulse durations: 210, 300, 400, 500, 600, 700, 800, 900, 1000, 2000, 5000, 10000 fs. In total, 1320 combinations of parameters (11 beam radii, 10 numbers of pulses per burst, 12 pulse durations) were tested, and 1320 cavities were milled.

### Laser beam characterisation

**Figure 2:**
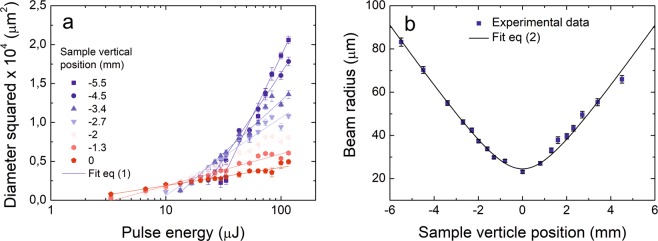
Gaussian beam size characterisation. (**a**) Beam radius calculation at different sample vertical positions *z* by damage diameter squared – versus pulse energy relationship, experimental data fitted by Eq. (1). (**b**) Gaussian beam radius *w*(*z*) dependence on sample vertical position *z*, fitted by beam propagation Eq. (2).

Beam radius at various *z* positions was measured by a well-known *D*^2^ technique, described in^18^. Some of the measurement results are depicted in Fig. 2a. The relationship between damage diameter squared *D*^2^ and pulse energy *E*_p_ is described by^18^:

1 $${{D}}^{2}=2{{w}}_{0}^{2}\,\mathrm{ln}(\frac{{{E}}_{p}}{{{E}}_{{\rm{th}}}}),$$

*E*_th_ – damage threshold energy. Therefore, at each *z* position, beam radii are extracted from the linear fit, as shown in Fig. 2b. The Gaussian-beam propagation equation^19^

2 $${w}({z})={{w}}_{0}{[1+{(\frac{({\boldsymbol{z}}-{{\boldsymbol{z}}}_{0}){\lambda }{{M}}^{2}}{{\pi }{{w}}_{0}^{2}})}^{2}]}^{\frac{1}{2}},$$

where *w*_0_ is the laser spot size in focus, *z*_0_ is the focal position of the beam, *λ* = 1030 nm is the wavelength of irradiation. The Eq. (2) was used to fit the experimental data, and beam quality parameter M^2^ = 1.09 ± 0.02 was retrieved, which coincidence well with the value provided by the laser manufacturer.

### Sample characterisation

**Figure 3:**
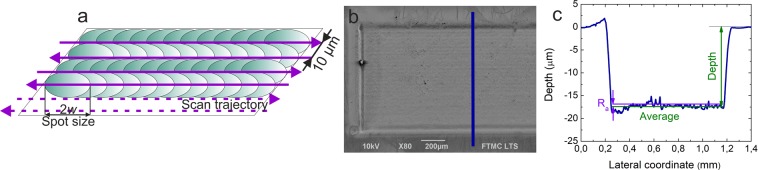
Copper sample processing scheme by laser and measurement by stylus profiler. (**a**) Visualisation of the scanning process during ablation of the rectangle cavity: each layer consisted of parallel lines, separated by the hatch distance of 10 µm; (**b**) SEM image of the ablated cavity with a line indicating the location of the stylus profiler measurement; (c) profile of the laser ablated cavity: the average depth of the cavity 17.6 µm, surface roughness *R*_a_ = 0.4 µm. Laser process parameters for (**b**) and (**c**): the number of pulses per burst *N* = 3, pulse duration *τ* = 10 ps, pulse fluence *F*_0_ = 3.2 J/cm^2^.

Copper samples with dimensions of 50 × 50 × 5 mm^3^, purity of 99.9% and surface roughness of R_a_ < 0.1 µm were used as a target material for laser ablation. Copper is a test metal widely used for the study of laser ablation characteristics. For sample visualisation, scanning electron microscope (SEM) (JSM-6490LV, JEOL) was used. Stylus profiler (Dektak 150, Veeco) was used to measure depths of the cavities (Fig. 3). The surface roughness *R*_*a*_ and cavity volume were extracted from the cavities profile measurements.

## Results and Discussion

### The number of pulses per burst

To find the real advantage of the burst regime, the beam size optimisation was introduced for each experiment with various numbers of pulses per burst. In general, a highly defocused laser beam should not remove the material from the target due to laser fluence being lower than the ablation threshold of the material. The peak pulse fluence *F*_0_ depends on the beam radius *w*_0_ as:

3 $${{F}}_{0}=\frac{2{{E}}_{{\boldsymbol{p}}}}{{\pi }{{w}}_{0}^{2}},$$

where *E*_p_ is the pulse energy of the individual pulses in the burst, not the energy of the whole burst. Similarly, peak pulse fluence refers to the fluence of the individual pulses in the burst. For the burst fluence, (3) should be multiplied by the number of pulses in the burst. Infinitely tightly focused laser beam would make a deep and narrow crater, but the volume of the removed material would maintain infinitely small. Therefore, the optimum point of the laser beam size for specific pulse energy, where the maximum volume of the material is removed, exists. This optimisation method allows finding at the same time, the highest ablation efficiency and highest ablation rate point for any combination of the processing parameters^4,17^. The mistake would be to optimise pulse energy to find the highest ablation efficiency and optimum fluence than use maximum average power from the laser to reach the highest ablation rate by increasing the spot size to maintain the optimum fluence. When the beam size on the sample surface is increased, the ablation threshold *F*_th_ is decreased due to the possibility for laser radiation to hit a defect^20,21^, meaning that the optimum fluence *F*_opt_ may shift down. According to the ablation model, the optimal and threshold fluences are interrelated^6^:

4 $${{F}}_{{\rm{opt}}}={{e}}^{2}{{F}}_{{\rm{th}}}.$$

**Figure 4:**
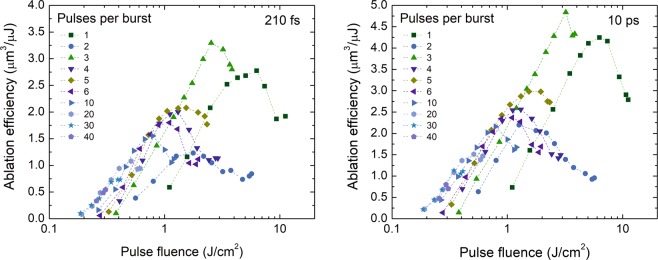
Ablation efficiency versus peak pulse fluence for a different number of pulses per burst and various pulse durations. Pulse fluence was varied by increasing the beam size. The laser wavelength *λ* = 1030 nm, burst repetition rate *f*_B_ = 300 kHz, intra-burst repetition rate *f*_P_ = 64.5 MHz, beam scanning speed *v* = 1 m/s. Graphs for all tested pulse duration can be found in the Supplementary Material.

The ablation efficiency has maxima at specific peak pulse fluence values. Experimentally it was found that the fluence values were dependent on the pulse number in the burst, but the pulse duration effect was less pronounced (see Fig. 4). The highest efficiency was measured for 3 pulses in the burst for all pulse durations. The increase in ablation efficiency compared with the single-pulse regime was from 11 to 20%, depending on the laser pulse duration used in experiments.

**Figure 5:**
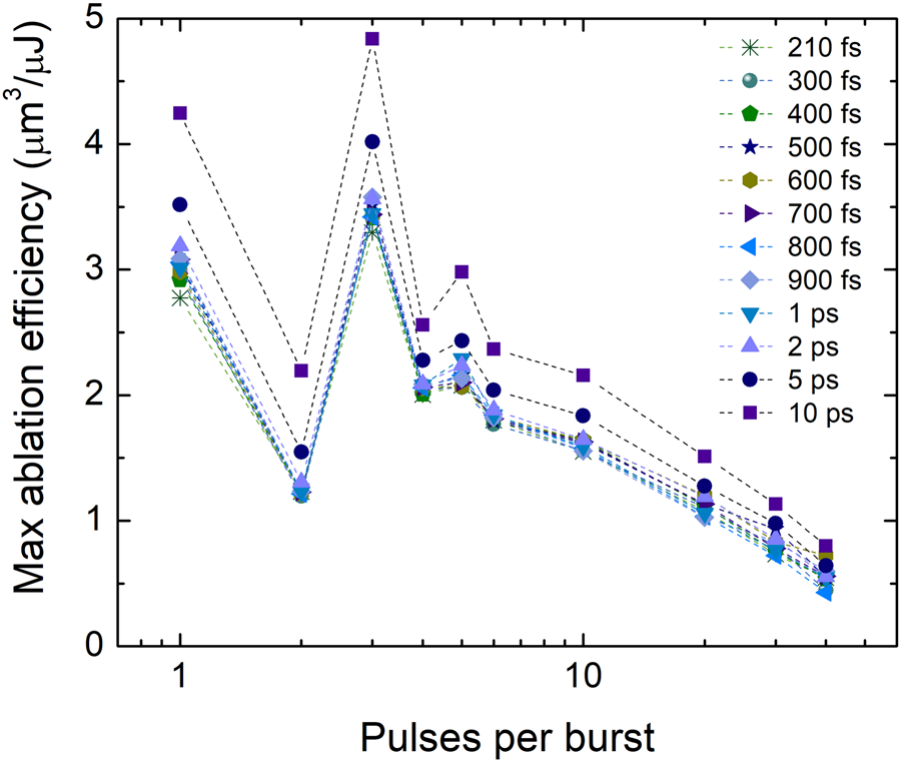
Maximum ablation efficiency versus the pulse number in the burst for different pulse durations. Data are extracted from the beam size optimisation experiments (Fig. 4). The laser wavelength *λ* = 1030 nm, burst repetition rate *f*_B_ = 300 kHz, intra-burst repetition rate *f*_P_ = 64.5 MHz, beam scanning speed *v* = 1 m/s.

The maximum ablation efficiencies at the optimum fluences were extracted from Fig. 4 and plotted versus the number of pulses in the burst (Fig. 5). The maximum ablation efficiency decreased sharply when 2 pulses in the burst were used, then suddenly grown up and exceeded the value of the single-pulse regime for 3 pulses per burst. For 4 pulses per burst, the maximum efficiency decreased again, for 5 pulses – marginally increased, later it decreased gradually. This behaviour was observed for all tested pulse durations. The explanation of the efficiency decrease for 2 pulses per burst and increase for 3 pulses per burst is discussed in^15^. The time interval after the first pulse hit the target material is as long as 15.5 ns and is not sufficient for ejected particles to fly away from the interaction area. As shown by the molecular dynamics simulation, particles ejected during laser ablation can move at ~2 km/s speed^22^, which results in a 31 µm distance after 15.5 ns. Therefore, the second pulse is attenuated by particle plume. Also, ablation products made by the first pulse are forced by the second pulse to be redeposited back on substrate^23^, which could lead to low ablation efficiency by the second laser pulse. Redeposited hot particles contribute to the ablation efficiency increase as hot material has a higher absorptance^24^. The calorimetric measurement for copper showed that absorptance for 3 pulses per burst is almost twice as high as a one for the single pulse regime^10^. Also, the higher temperature of the interaction area might improve the ablation efficiency as the energy required to evaporate the material by the third pulse is lower^25^. The advantage of using the burst regime in the case of ablation efficiency was only visible for the regime with 3 pulses in the burst. For all other pulse numbers in the burst, the efficiency was lower compared to the single-pulse regime.

The highest ablation efficiency of 4.8 µm^3^/µJ and ablation rate of 10.5 mm^3^/min was reached in this work for 3 pulses per burst and 10 ps pulse duration regime at the 120 µJ burst energy. To our best knowledge, this is the highest laser milling ablation efficiency obtained for copper material by ultrashort pulse lasers emitting at ~1 µm optical wavelength. The higher ablation efficiency of ~7.6 µm^3^/µJ was demonstrated only by punching (ablation of a crater) with the ablation-cooled material removal (burst of pulses *N* = 800, intra-burst repetition rate of *f*_P_ = 3456 MHz, burst repetition rate *f*_B_ = 1 kHz, laser wavelength *λ* = 1040 nm, pulse duration of *τ* = 1 ps)^2^. Due to the low burst repetition rate of *f*_B_ = 1 kHz, the ablation rate was only 0.03 mm^3^/min. As discussed in^26^, the laser milling has lower ablation efficiency compared to the laser punching efficiency of ~10 times due to differences in heat accumulation and melt flow in two machining approaches. The ablation-cooling regime was also investigated for copper with a high-power laser (burst repetition rate of *f*_B_ = 200 kHz, burst energy *E*_B_ = 93 µJ). In this case, the ablation rate as high as 6 mm^3^/min was reached for punching with *N* = 560 pulses in the burst, with *f*_P_ = 1600 MHz intra-burst repetition rate^27^. However, it was still 40% lower than the ablation rate for laser milling achieved in our study. In paper^28^, the punching-mode processing in the burst regime was utilised for copper and efficiency reached ~3.2 µm^3^/µJ by *N* = 5, *f*_B_ = 50 MHz, *λ* = 1064 nm, *τ* = 10 ps. A lot of work in the pulse energy optimisation for bursts was done by Neuenschwander *et al*., who found the maximum milling ablation efficiency for copper of 2.6 µm^3^/µJ^8,15,29^ or 2.9 µm^3^/µJ^7^ for *λ* = 1064 nm and *λ* = 1030 nm which is more than 40% lower than the ablation efficiency achieved in our study. The beam-size-optimised single-pulse regimes showed the milling ablation efficiency of copper as high as 2.5 µm^3^/µJ for *λ* = 1064 nm and *τ* = 10 ps^4,17^.

### Pulse duration

**Figure 6:**
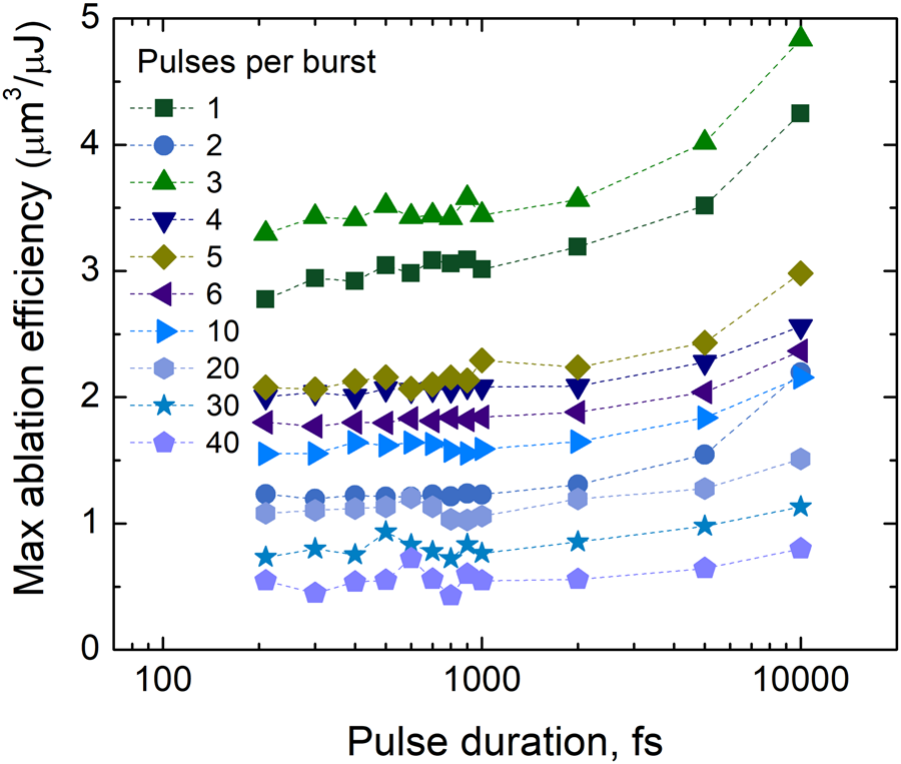
The maximum ablation efficiency for different numbers of pulses per burst versus pulse duration. The laser wavelength *λ* = 1030 nm, burst repetition rate *f*_B_ = 300 kHz, intra-burst repetition rate *f*_P_ = 64.5 MHz, beam scanning speed *v* = 1 m/s.

The maximum ablation efficiencies at the optimum fluences were extracted from Fig. 4 and graph versus pulse duration is plotted in Fig. 6. The increase in the maximum ablation efficiency at longer pulse durations was observed for all number of pulses in the burst. For 3 pulses per burst, the maximum ablation efficiency was 32% higher at the 10 ps pulse duration compared to 210 fs.

### Surface roughness

**Figure 7:**
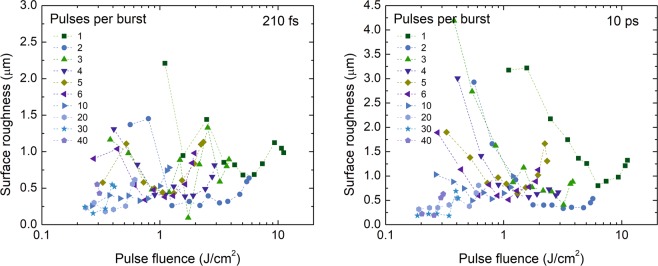
The surface roughness of the bottom of the laser-ablated cavity versus peak pulse fluence for a various number of pulses per burst and various pulse durations. The laser wavelength *λ* = 1030 nm, burst repetition rate *f*_B_ = 300 kHz, intra-burst repetition rate *f*_P_ = 64.5 MHz, beam scanning speed *v* = 1 m/s. Graphs for all tested pulse durations can be found in see Supplementary Material.

The lowest cavity surface roughness was obnained in the same range of pulse fluences (1–3 J/cm^2^) as the highest ablation efficiency (see Fig. 7). This means that two highly important micro-machining characteristics: the ablation efficiency and surface quality can be optimised simultaneously. For the single-pulse regime, the surface roughness was always higher compared to the burst mode – another advantage of the burst regime.

**Figure 8:**
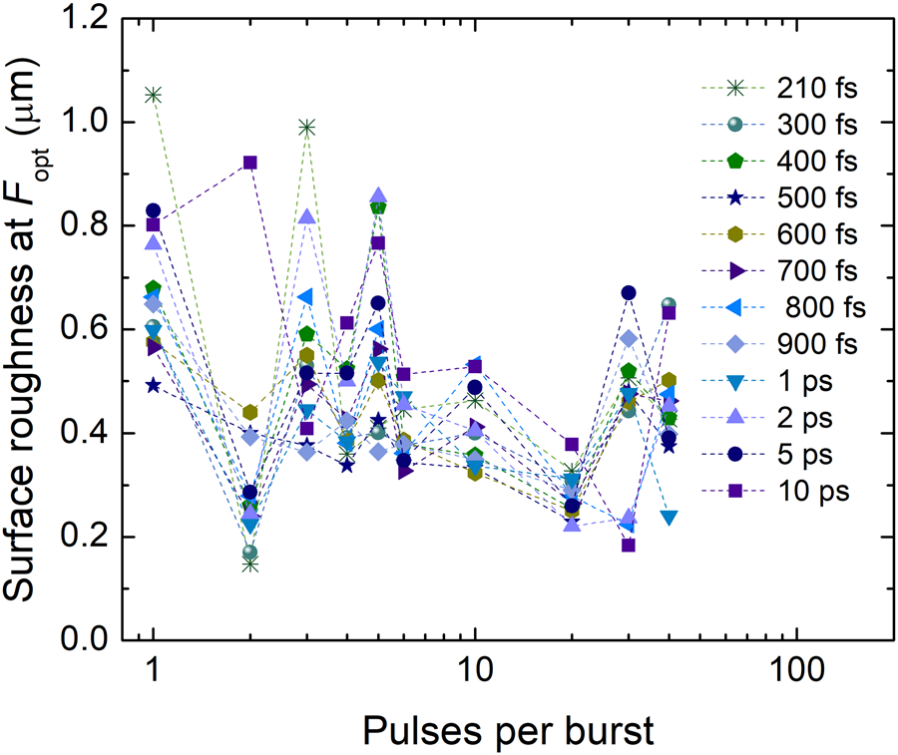
Surface roughness at the optimum fluence for the highest ablation efficiency versus the number of pulses per burst and various pulse durations. The laser wavelength *λ* = 1030 nm, burst repetition rate *f*_B_ = 300 kHz, intra-burst repetition rate *f*_P_ = 64.5 MHz, beam scanning speed *v* = 1 m/s.

The surface roughness at the optimum laser fluence *F*_opt_ for the highest ablation efficiency was extracted from Fig. 7 and plotted versus the number of pulses per burst (Fig. 8). The surface roughness as small as 0.4 µm was achieved for the most efficient regime of 3 pulses per burst and pulse duration of 10 ps.

### Laser milling of complex 3D cavities

**Figure 9:**
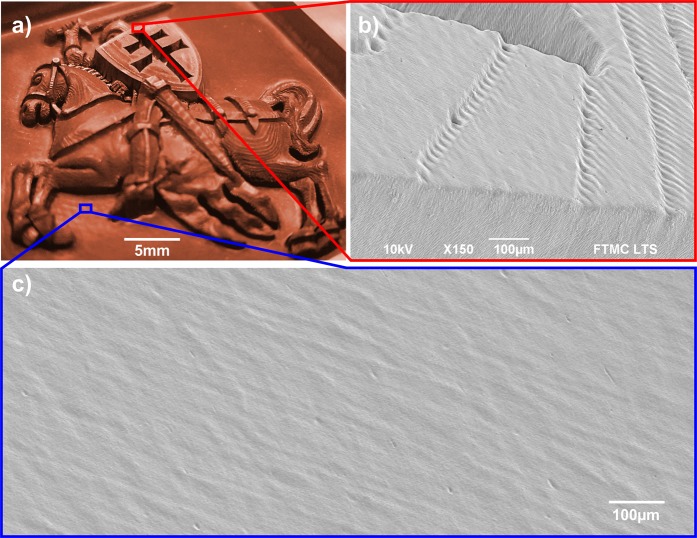
Example of efficient laser milling. (**a**) Optical image of the coat of arms of Lithuania milled in copper plate. (**b**) SEM image of laser milled surface illustrating layer-by-layer removal. (**c**) SEM image of the bottom surface of the laser-milled cavity. Laser parameters - 3 pulses per burst, laser wavelength *λ* = 1030 nm, burst repetition rate *f*_B_ = 300 kHz, intra-burst repetition rate *f*_P_ = 64.5 MHz, beam scanning speed *v* = 1 m/s.

The burst mode micro-machining quality was tested by milling complex 3D cavities (Fig. 9). The 3D micro-machining was realised using a layer-by-layer removal technique starting from the top of the sample. The distance between the focusing lens and sample surface was adjusted after each layer removal to maintain the optimal beam width on the ablated surface (see Fig. 9b). The most efficient 3 pulses per burst regime was selected, and the pulse duration was set to 10 ps. High-quality complex 3D cavities were laser milled. No side effects or melting was seen for longer pulses. Pulses of 10 ps length were short enough to minimise melt formation on the copper surface, which coincidence well with electron-ion thermalisation time in copper^30^. The bottom of the cavities was smooth, with no bumps or unwanted parasitic structure formation, which could ruin the aesthetic appearance of the cavities (see Fig. 9c).

## Conclusions

The study of copper laser ablation by pulses of 64.5 MHz intra-burst pulse repetition rate revealed that, in the best case, the ablation efficiency could be improved by 20% compared to the single-pulse regime for the beam-size-optimised regimes. All other numbers of pulses per burst demonstrated lower ablation efficiency. To our best knowledge, the beam-size-optimised, 3 pulses per burst processing let us reach the highest ever published laser milling ablation efficiency of copper by ultrashort pulses −4.8 µm^3^/µJ. Another advantage of the burst mode compared to the single-pulse regime ablation was lower surface roughness of the bottom of the ablated cavities. The lowest surface roughness achieved by the single-pulse regime was several times higher than that measured for the burst mode. In the range of pulse duration between 210 fs and 10 ps, the ablation efficiency increased by 32% for longer pulses, and micro-machining quality improved. In conclusion, the usage of bursts of pulses for laser micro-processing of copper is advantageous only when 3 pulses per burst are used – the ablation efficiency and quality are increased compared to the single-pulse regime.

## Supplementary information

Highly-efficient laser ablation of copper by bursts of ultrashort tuneable (fs-ps) pulses
