# Supplementary material for: Highly-efficient laser ablation of copper by bursts of ultrashort tuneable (fs-ps) pulses
Source: Sci Rep. 2019 Aug 22;9:12280. doi: 10.1038/s41598-019-48779-w (PMC6706424; doi:10.1038/s41598-019-48779-w)
Supplement: Supplementary file 1 — Highly-efficient laser ablation of copper by bursts of ultrashort tuneable (fs-ps) pulses [file 41598_2019_48779_MOESM1_ESM.pdf]

Supplementary material

Highly-efficient laser ablation of copper by bursts of ultrashort tuneable (fs-ps) pulses

Andrius Žemaitis[1], Paulius Gečys[1], Martynas Barkauskas[2], Gediminas Račiukaitis[1] and Mindaugas Gedvilas[1]

[1] Center for Physical Sciences and Technology, Savanoriu Ave. 231, LT-02300 Vilnius, Lithuania

[2] Light Conversion Ltd., Keramiku st. 2B, LT-10233 Vilnius, Lithuania

Correspondence and requests for materials should be addressed to A.Ž. (E-mail: andrius.zemaitis@ftmc.lt).

Number of pulses per burst

The ablation efficiency has maxima at specific laser fluence values. The fluence values were found to be dependent on the pulse number in the burst, but almost independent on the pulse duration (Fig. 1). The highest efficiency was measured for 3 pulses in the burst for all pulse durations. The increase in the ablation efficiency comparing to the single-pulse regime was from 11 to 20 %, depending on pulse duration. The shift of the curves to lower fluence is evident with increasing the number of pulses in the burst.

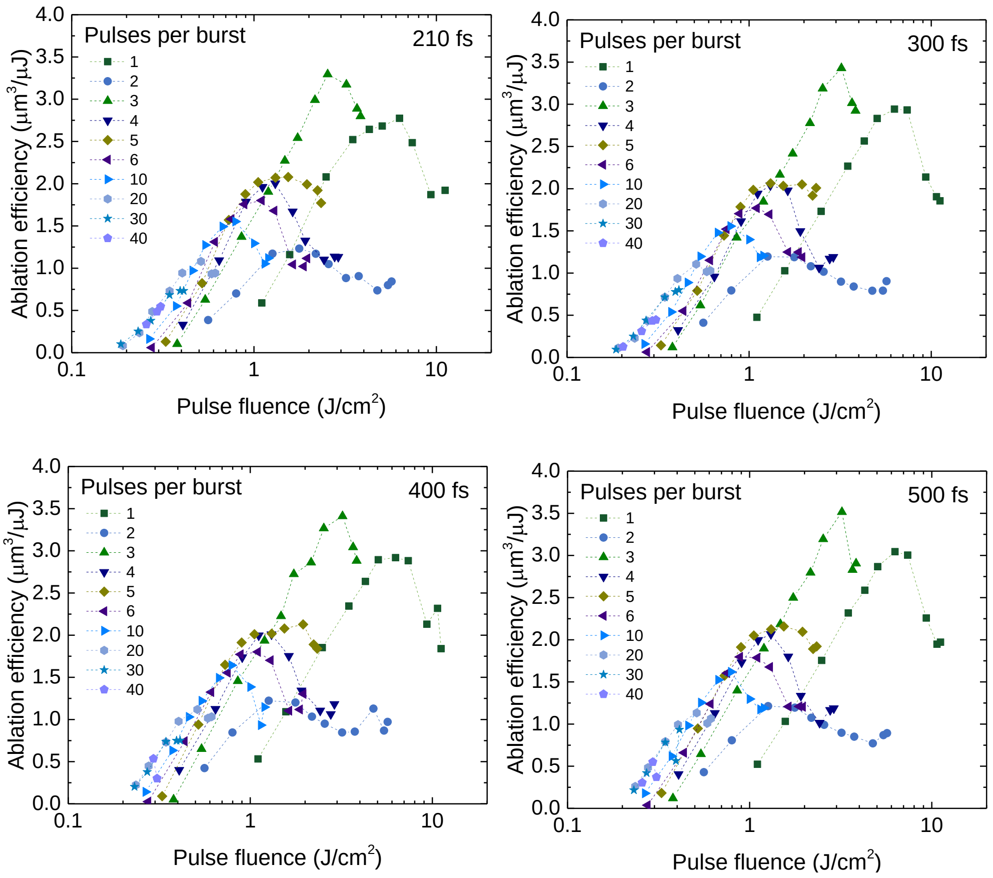

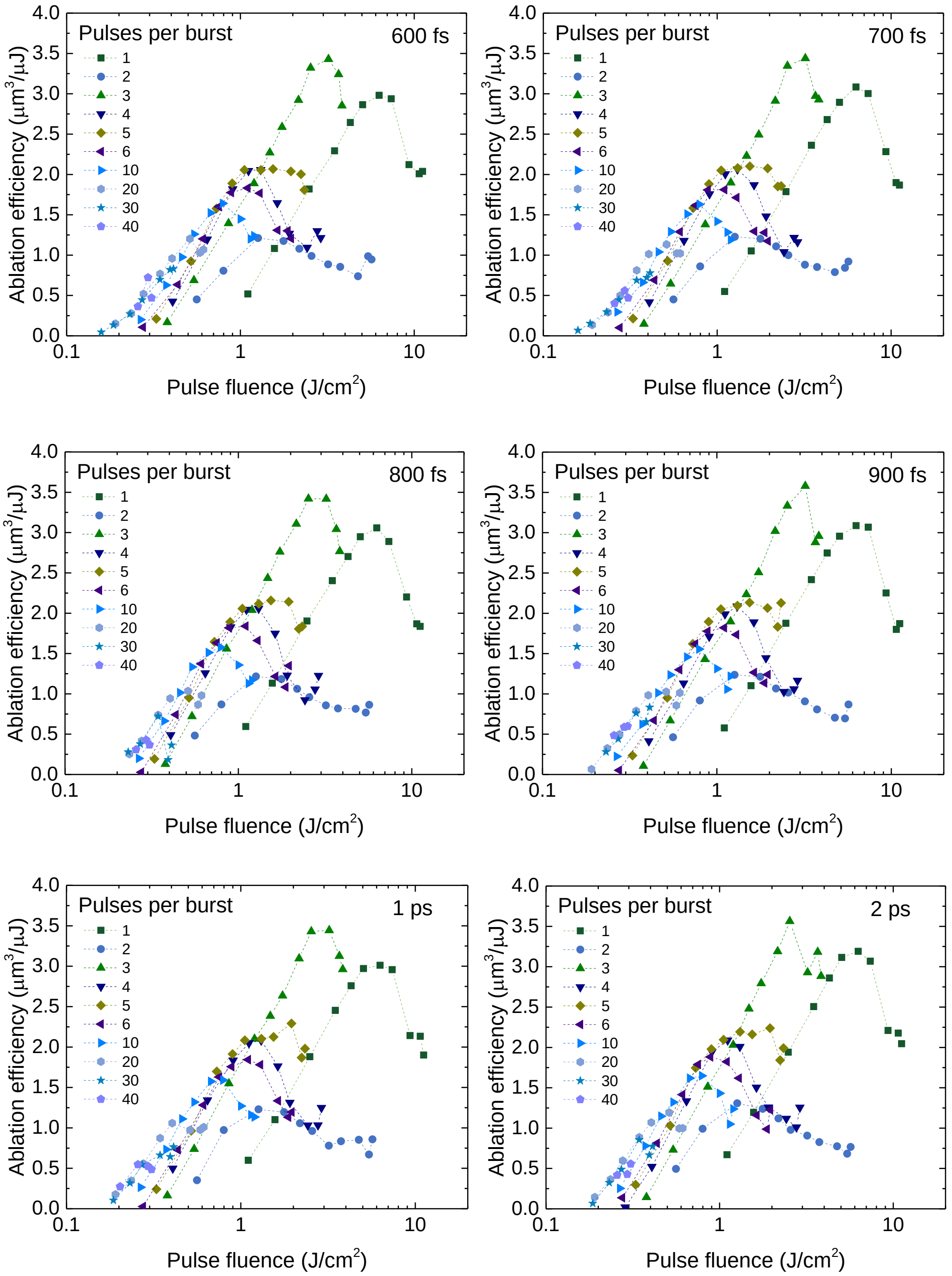
Pulses per burst
600 fs
700 fs
800 fs
900 fs
1 ps
2 ps
1
2
3
4
5
6
10
20
30
40
Ablation efficiency (µm³/µJ)
Pulse fluence (J/cm²)
0.0
0.5
1.0
1.5
2.0
2.5
3.0
3.5
4.0
0.1
1
10

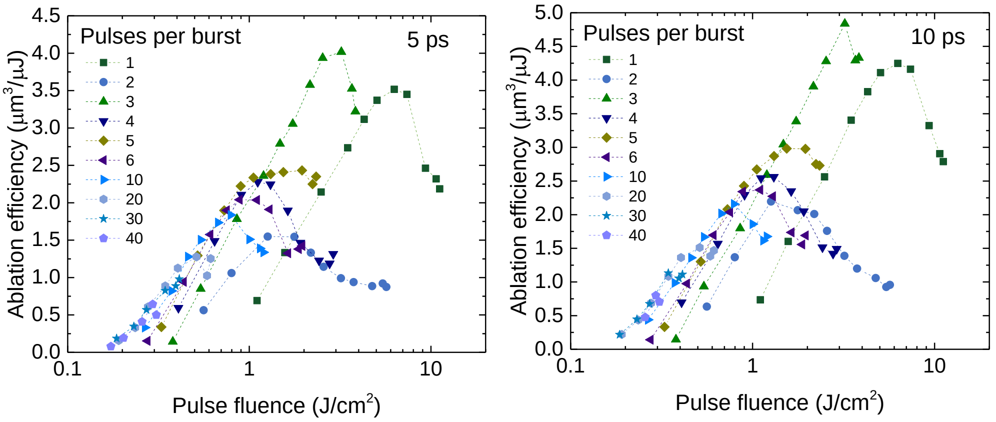

Fig. 1. Ablation efficiency versus peak pulse fluence for various numbers of pulses per burst and different pulse durations. The laser fluence was changed by increasing the beam size. The laser wavelength λ = 1030 nm, burst repetition rate f_B = 300 kHz, intra-burst repetition rate f_P = 64.5 MHz, beam scanning speed v = 1 m/s.

Surface roughness

The surface roughness on the bottom of the cavities was lowest at laser fluences in the same range, which was found for the highest ablation efficiency, of 1-3 J/cm² (Fig. 2).

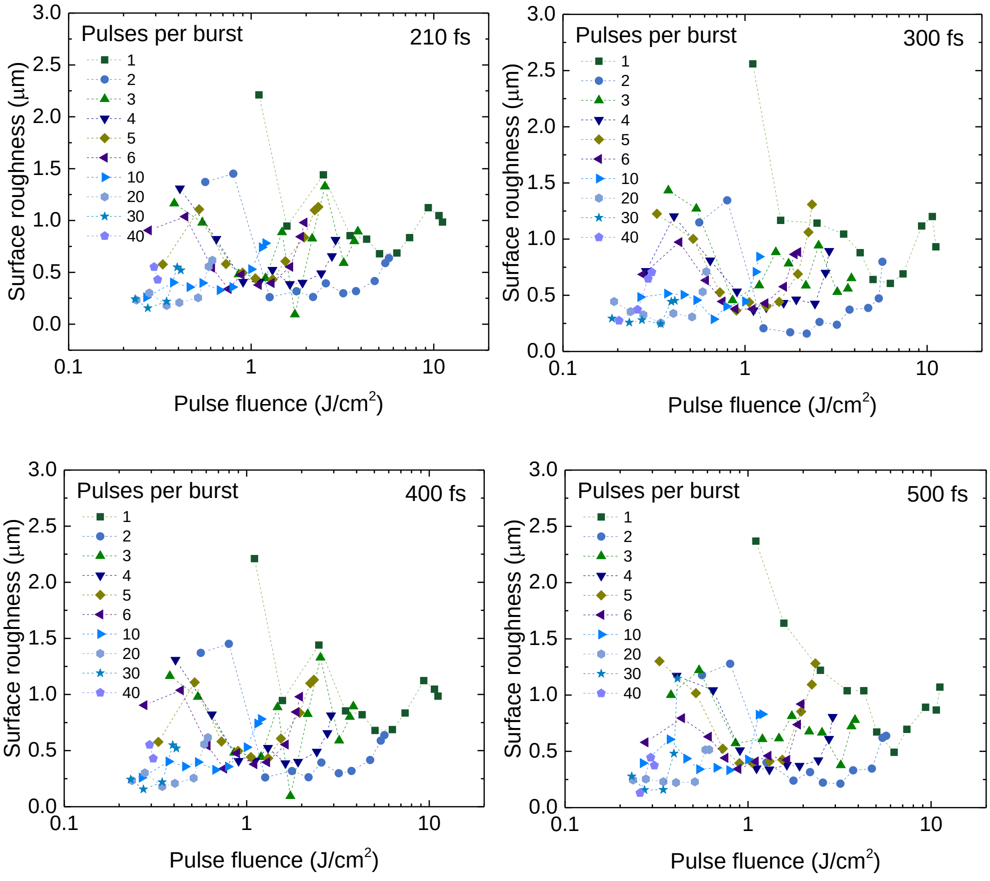

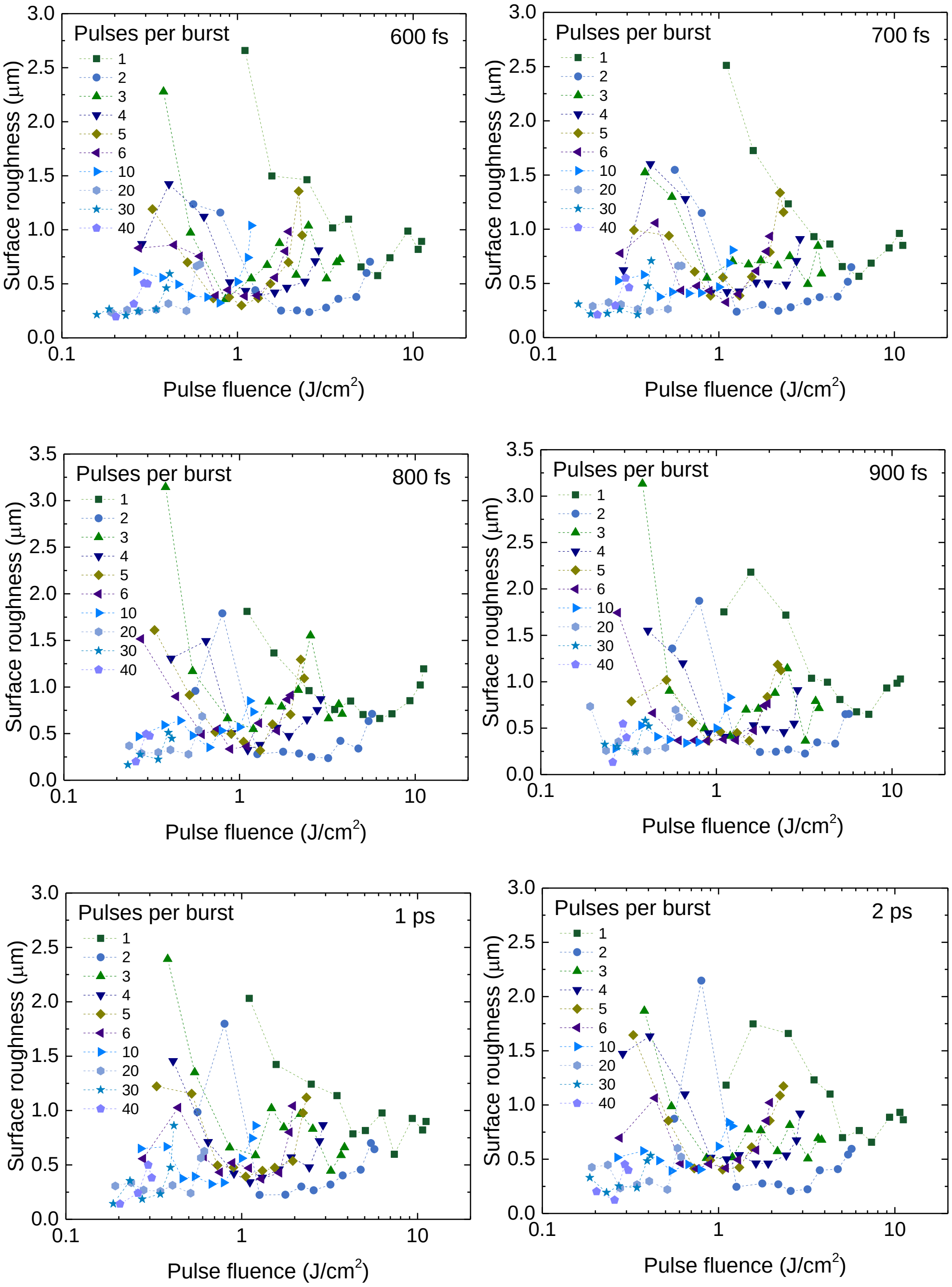
Pulses per burst
600 fs
1
2
3
4
5
6
10
20
30
40
Surface roughness (μm)
Pulse fluence (J/cm²)
0.0
0.5
1.0
1.5
2.0
2.5
3.0
0.1
1
10
Pulses per burst
700 fs
Surface roughness (μm)
Pulse fluence (J/cm²)
Pulses per burst
800 fs
3.5
Surface roughness (μm)
Pulse fluence (J/cm²)
Pulses per burst
900 fs
Surface roughness (μm)
Pulse fluence (J/cm²)
Pulses per burst
1 ps
Surface roughness (μm)
Pulse fluence (J/cm²)
Pulses per burst
2 ps
Surface roughness (μm)
Pulse fluence (J/cm²)

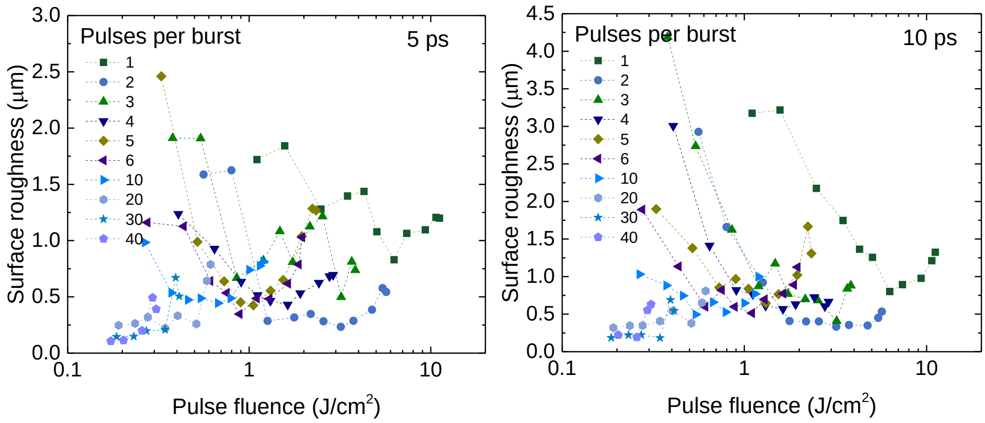

Fig. 2 Surface roughness on the bottom of the cavity versus peak pulse fluence for various numbers of pulses per burst and various pulse durations. The laser wavelength λ = 1030 nm, burst repetition rate f_B = 300 kHz, intra-burst repetition rate f_P = 64.5 MHz, beam scanning speed v = 1 m/s.
